# Multi-polygenic score approach to trait prediction

**DOI:** 10.1038/mp.2017.163

**Published:** 2017-08-08

**Authors:** E Krapohl, H Patel, S Newhouse, C J Curtis, S von Stumm, P S Dale, D Zabaneh, G Breen, P F O'Reilly, R Plomin

**Affiliations:** 1 MRC Social, Genetic and Developmental Psychiatry Centre, Institute of Psychiatry, Psychology and Neuroscience, King’s College London, London, UK; 2 Department of Biostatistics and Health Informatics, Institute of Psychiatry, Psychology and Neuroscience, King’s College London, London, UK; 3 NIHR Biomedical Research Centre at South London and Maudsley NHS Foundation Trust and King’s College London, London, UK; 4 Farr Institute of Health Informatics Research, UCL Institute of Health Informatics, University College London, London, UK; 5 Department of Psychology, Goldsmiths University of London, New Cross, London, UK; 6 Department of Speech and Hearing Sciences, University of New Mexico, Albuquerque, NM, USA

## Abstract

A primary goal of polygenic scores, which aggregate the effects of thousands of trait-associated DNA variants discovered in genome-wide association studies (GWASs), is to estimate individual-specific genetic propensities and predict outcomes. This is typically achieved using a single polygenic score, but here we use a multi-polygenic score (MPS) approach to increase predictive power by exploiting the joint power of multiple discovery GWASs, without assumptions about the relationships among predictors. We used summary statistics of 81 well-powered GWASs of cognitive, medical and anthropometric traits to predict three core developmental outcomes in our independent target sample: educational achievement, body mass index (BMI) and general cognitive ability. We used regularized regression with repeated cross-validation to select from and estimate contributions of 81 polygenic scores in a UK representative sample of 6710 unrelated adolescents. The MPS approach predicted 10.9% variance in educational achievement, 4.8% in general cognitive ability and 5.4% in BMI in an independent test set, predicting 1.1%, 1.1%, and 1.6% more variance than the best single-score predictions. As other relevant GWA analyses are reported, they can be incorporated in MPS models to maximize phenotype prediction. The MPS approach should be useful in research with modest sample sizes to investigate developmental, multivariate and gene–environment interplay issues and, eventually, in clinical settings to predict and prevent problems using personalized interventions.

## Introduction

Genome-wide association studies (GWASs) have been successful in identifying thousands of associations for hundreds of complex traits and common disorders.^1^ One use of GWAS results is to understand biological pathways between genotypes and phenotypes. Another use, the focus of the present research, is to estimate genetic propensities of individuals to predict individuals’ future problems and potential and, eventually, to develop personalized interventions that meet individual medical, psychiatric and educational needs. Both goals have been hindered by the ubiquitous GWA finding that the largest effect sizes are extremely small.^2^ For example, the largest population effect sizes found for common variants in height or body mass index (BMI) account for only ~1% of the variance.^3, 4^ We know empirically that the vast majority of common genetic variants for most traits have a markedly lower effect than 1%.^2^

The highly polygenic nature of complex traits and common disorders poses an immense challenge for understanding the biological mechanisms linking single variants with phenotypes. However, when the priority is phenotypic prediction, polygenic scores can be used to aggregate the effects of many DNA variants in order to investigate their joint predictive power.^5, 6^ Rather than just using single-nucleotide polymorphisms (SNPs) that reach genome-wide significance, a recent development is to aggregate a much larger number of SNPs, weighted by their GWA effect size estimate, as long as together they increase the prediction in an independent sample, even if some SNPs have no real effect.^7^ For example, for height, a polygenic score that aggregates the effects of ~2000 SNPs accounts for 21% of the variance of height in independent samples.^3^

The other defining characteristic of complex traits and common disorders is the abundance of genetic correlations between them. There is consistent evidence for genetic correlations between psychiatric disorders, between anthropometric traits and between educational and cognitive traits, as well as for genetic correlations across these categories.^8, 9, 10, 11^

Genetic correlation can arise from pleiotropy, the phenomenon of multiple traits being associated with the same gene or genetic variant.^8^ Genetic correlation can also reflect shared biological pathways or more indirect linkage.^12^ Regardless of its cause, genetic correlation between different traits means that a polygenic score based on one trait can predict a different outcome trait, with predictive accuracy a function of the shared genetic signal between them. Therefore, when the aim is prediction, genetic correlation can be exploited for trait prediction while remaining agnostic to the underlying mechanisms.

A primary goal of polygenic scores, which aggregate the effects of thousands of trait-associated genetic variants discovered in GWAS, is to estimate individual-specific genetic propensities. This is typically achieved using a single polygenic score, but here we use an approach to increase predictive power by exploiting the joint power of multiple discovery GWASs. We use a multi-polygenic score (MPS) approach that exploits genetic correlations between the outcome trait and a multitude of traits by using the joint predictive power of multiple polygenic scores in one regression model.

We selected GWASs from a centralized repository of summary statistics—based on their statistical power and regardless of prior evidence for association with the outcomes—to predict three core developmental outcomes in our independent target sample: educational achievement, BMI, and general cognitive ability. Using repeated cross-validation, we trained and validated the prediction models using elastic net regularized regression, a multiple regression model suited to deal with a large number of correlated predictors while preventing overfitting.^13^ We subsequently tested how well these models predict outcomes in an independent test set.

Here, we employ a MPS approach that uses publicly available GWAS summary statistics to estimate individual-level genetic propensities and predict developmental outcomes in an independent target sample. This stands in contrast to multi-trait approaches that rely on access to individual-level data in the discovery data sets because they make use of a method from animal breeding in which the total genetic effect (‘breeding value’) of each individual in a discovery data set is estimated from the best linear unbiased predictor in a multi-trait random-effects model that can be used for individual-level prediction in the validation data sets. These multi-trait methods are not applicable to GWAS summary statistics when genotype data are unavailable because of privacy or logistical constraints that are frequently the case.

The declared aim of the current MPS approach is to maximize prediction of developmental outcomes, rather than investigating their etiology. This stands in contrast to multi-trait meta-analytic approaches of GWAS summary statistics that relies on substantial and consistent correlations between discovery GWASs and whose main aim is variant discovery.^14, 15, 16, 17^ The current MPS approach allows for, but does not require, correlation among polygenic predictors.

## Materials and methods

### Sample

The target sample comprised genome-wide SNP and phenotypic data from 6710 unrelated adolescents drawn from the UK representative Twins Early Development Study (TEDS). TEDS is a multivariate longitudinal study that recruited over 11 000 twin pairs born in England and Wales in 1994, 1995 and 1996. Both the overall TEDS sample and the genotyped subsample have been shown to be representative of the UK population.^18, 19, 20^ The project received approval from the Institute of Psychiatry ethics committee (05/Q0706/228) and parental consent was obtained before data collection. We processed the genotypes for the 6710 individuals using stringent quality control procedures followed by imputation of SNPs using the Haplotype Reference Consortium reference panel^21^ (Supplementary Methods S1).

### Predictors

#### Discovery data sets: GWAS summary statistics

We selected GWAS summary statistics from *LD hub*, a centralized repository for summary statistics^22^ based on their statistical power—regardless of prior evidence for association with our outcome traits. Specifically, we included 81 GWAS summary statistics that were either publically downloadable or obtained via correspondence and had a linkage disequilibrium (LD) score^23^ heritability *z*-score >5, indexing good statistical power (which is a function of variance explained and sample size). Supplementary Table S1 provides details of all GWAS summary statistics included in our analyses.

The published version of the child IQ GWAS included the present target sample of TEDS. Therefore, to avoid bias, the present analyses used summary statistics from a rerun of the GWAS meta-analysis excluding TEDS.

#### Polygenic scores

We created 81 genome-wide polygenic scores for each of the 6710 individuals in the TEDS sample using summary statistics from the GWAS described above (Supplementary Table S1). After quality control (Supplementary Methods S1), the study data included 7 581 516 genotyped or well-imputed (info >0.70) SNPs. These were quality controlled and coordinated with each of the summary statistics, respectively, by excluding markers due to nucleotide inconsistencies or low minor allele frequency (<1%). Number of markers before and after quality control and coordination with the study data are listed in Supplementary Table S1.

We constructed polygenic scores as the weighted sums of each individual’s trait-associated alleles across all SNPs. We used LDpred^24^ to construct the scores. LDpred uses a prior on the markers’ effect sizes and adjusts summary statistics for LD between markers. Scores were standardized and adjusted for 30 principal components. More details on the construction of the polygenic scores are provided in Supplementary Methods S2.

### Outcomes

To illustrate the MPS approach, we selected three key developmental outcomes:

Educational achievement operationalized as the mean grade of the three compulsory subjects (Mathematics, English and Science) attained on the standardized United Kingdom General Certificate of Secondary Education (GCSE), taken by almost all (>99%) pupils at the end of compulsory education at age 16 years.

General cognitive ability at age 12 years assessed by two verbal and two nonverbal cognitive standardized tests.

BMI at age 9 years that was age and sex adjusted using external reference data.

Supplementary Methods S3 and Figure S1 contain detailed descriptions of the three measures.

### Models

#### Single-polygenic score models

To estimate the separate prediction of each predictor, we fit a series of simple linear regression models for each of the 81 polygenic scores and each of the 3 outcomes. For each GWAS-outcome combination, three models were run using polygenic scores created with Gaussian mixture weights of 1, 0.1 and 0.01, respectively. The model that explained the most variance in the outcome (that is, largest cross-validated *R*^2^ in training data) was then entered into the multi-score model. These simple linear regression models were fit and validated in repeated 10-fold cross-validation (see section below for details) using the *lm* function implemented within the *caret* R package.^25^ Based on consistent evidence for extensive genetic correlations across complex traits and disorders, rather than summing up, the predictions of the single-score models were expected to substantially overlap.

#### MPS models

We used the MPS model to estimate the joint prediction of the 81 polygenic scores as well as the ranking of predictors by the magnitude of their contribution to predicting the outcome.

Conventional multiple linear regression models in the presence of a large number of predictors are subject to overfitting, and stepwise regression suffers from upward-biased coefficients and *R*^2^ (see, for example, Tibshirani^26^). We used elastic net regularized regression^13^ to predict outcomes and by selecting predictors and estimating their contribution to the prediction. Regularized regression models are general linear models that employ strict penalties to prevent overfitting. Elastic net allows for estimating the joint predictive ability of a large number of variables while preventing overfitting. Elastic net uses a linear combination of two regularization techniques, L2 regularization (used in ridge regression) and L1 regularization (used in LASSO (least absolute shrinkage and selection operator)) by simultaneously implementing variable selection (that is, dropping/retaining variables) and continuous shrinkage (that is, penalizing coefficients for overfitting); and it efficiently deals with multicollinearity by selecting or dropping groups of correlated variables.^13, 27^

Elastic net overcomes the limitation of LASSO that tends to select one variable from a group of correlated predictors and to ignore the others. *In situations* where predictors are non-independent or correlated (for example, sharing genetic signal or discovery cohorts) the elastic net has the advantage of including automatically all the highly correlated variables in the group (*grouping effect*).^13, 27, 28^

Final model coefficients are analogous to a conventional multiple linear regression output that allows for a ranking of predictors by the magnitude of their contribution to predicting the outcome. Overall variance explained by the model is indexed by the coefficient of determination, *R*^2^.

We used *glmnet* R package^15, 16, 17^ implemented within *caret* R package^25^ to conduct a series of linear elastic net regularized regressions and select polygenic predictors leading to an optimized final model for each outcome. Elastic net regularized regression employs two hyperparameters, alpha and lambda.^13^ As recommended to achieve optimized balance between variance explained and minimum bias, we fit models to tune over both alpha and lambda parameter values in repeated 10-fold cross-validation.^29^.

### Model training and testing

Generally, a predictive model is considered powerful when the model is capable of predicting outcomes in ‘unseen’ data with high accuracy. The performance of a model can therefore be evaluated by testing how well it predicts phenotypes of individuals whose data were not included in the construction of the prediction model.

Each model described in the preceding section was trained and tested using the following three-step strategy:

1. *Data splitting*. We randomly split the data set into a separate training set and test set (60% *train*, 40% *test*).
2. *Model training*. We used repeated cross-validation on the training set to train and optimize the model via validation.
3. *Model testing and comparison.* We applied the final model to the independent test set to obtain an unbiased estimate of model performance.

#### Model training

The training set was used to train and validate the model, this included hyperparameter tuning for the elastic net models. In order to optimize the balance between variance explained and minimum bias, we tested each model in 10-fold cross-validation with resampling.^29^ We split the training data randomly into 10 equal-sized subsets, using 9 subsets to train the model and the remaining subset as validation. The cross-validation process was repeated 10 times, with each of the 10 subsamples used once as the validation data.

Although cross-validation has been shown to produce nearly unbiased estimates of accuracy, variability of these estimates can be reduced by bootstrap methods, wherein available data are repeatedly sampled with replacement in order to mimic the drawing of future random sampling.^30, 31^ Therefore, to minimize variation across validation data sets, we repeated the 10-fold cross-validation 100 times with random data set partitions.^32^

The optimized or ‘final’ model is chosen based on the largest performance value (or smallest mean squared error). Predictors retained within the model and standardized coefficients index whether, and to what extent, they contribute to predicting the outcome. Model performance for the repeated cross-validation in the training set was summarized as mean-cv-*R*^2^_train_ from the resampling distribution.

#### Model testing and comparison

To obtain unbiased estimates of model performance, we used the parameters from the final model obtained from the repeated cross-validation in the training set to predict outcomes (that is, educational achievement, BMI and general cognitive ability) in the independent test set. To index prediction accuracy, we used the coefficient of determination, in the following referred to as *R*^2^_test_. Differences between mean-cv-*R*^2^_train_ and *R*^2^_test_ provide an index of out-of-sample error.

We used permutation to test the statistical significance of the difference in predictions between the MPS and the best single-score model. To test the null hypothesis of exchangeability of models, *H*_0_: _MPS_*R*^2^_test_=_best-single-score_*R*^2^_test_, we compared the observed _diff_*R*^2^_test_ (_MPS_*R*^2^_test_ – _best-single-score_*R*^2^_test_) against an empirical null distribution of no difference in predictions between the MPS and the best single-score model. We tested the exchangeability of models by randomly selecting either the MPS or the best single-score model to generate predictions. We then calculated the difference in *R*^2^ for two models with shuffled predictions. The process was repeated 100 000 times, generating an empirical null distribution of _diff_*R*^2^ under exchangeability of model predictions.

If the null hypothesis of no difference between models is true, it would not matter if we randomly exchange the model used for generating predictions. However, if the observed _diff_*R*^2^_test_ value falls outside of those obtained when randomly exchanging models, this represents evidence against the null hypothesis of no difference in prediction between models. The statistical significance, as expressed in an empirical *P*-value, is calculated as the fraction of permutation values that are at least as extreme as the original _diff_*R*^2^_test_ statistic observed in nonpermuted data.

## Results

### MPS predictions

The MPS models showed better prediction in the independent test set than the best single-score models. The best single-score models were the large 2016 GWAS of years of education predicting 9.8% of the variance in educational achievement and 3.6% in general cognitive ability in the test set. For BMI, Obesity class 1 achieved the best single-score prediction, explaining 3.8% of the variance. (See Supplementary Table S2 for full single-score models results; see Supplementary Figure S2 for a visual overview of the single-score model results.) The MPS models explained 10.9% variance in educational achievement, 4.8% in cognitive ability and 5.4% in BMI in the test set. The improvement in variance explained compared with the best single-score models was 1.1% (*P*=4e−03), 1.1% (*P*=2e−03) and 1.6% (*P*=1e−04), respectively.

**Figure 1:**
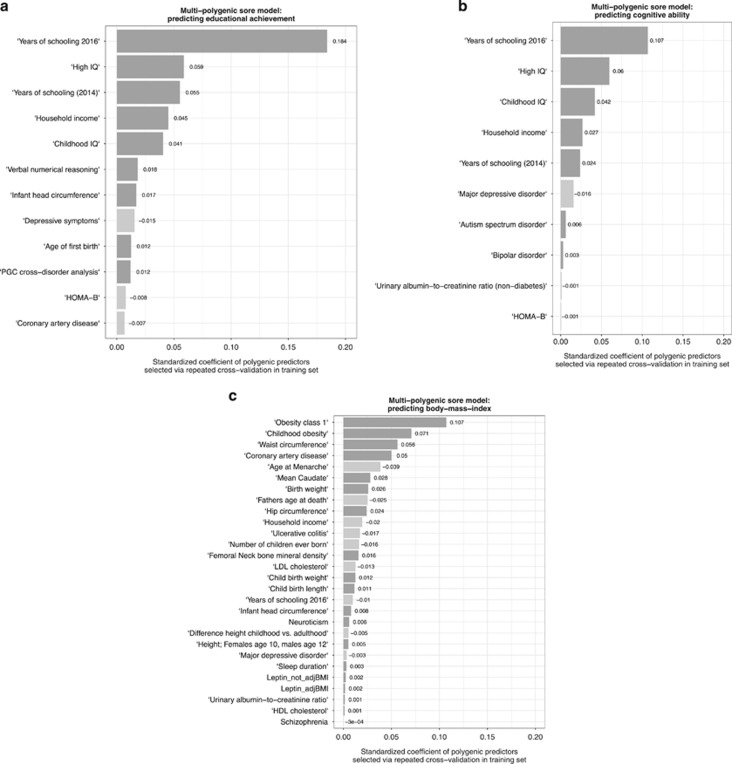
(**a**) Multi-polygenic score (MPS) model predicting educational achievement. Standardized coefficients of polygenic predictors selected by elastic net via repeated cross-validation in training set. Analogous to conventional multiple regression, a standardized coefficient represents the contribution of the predictor to the outcome when adjusting for all other variables in the model. The mean variance explained of the resampling distribution from the cross-validation was mean-cv-*R*^2^_train_=0.12. The out-of-sample prediction of the model was *R*^2^_test_=0.109. (**b**) MPS model predicting general cognitive ability. Standardized coefficients of polygenic predictors selected by elastic net via repeated cross-validation in training set. Analogous to conventional multiple regression, a standardized coefficient represents the contribution of the predictor to the outcome when adjusting for all other variables in the model. The mean variance explained of the resampling distribution from the cross-validation was mean-cv-*R*^2^_train_=0.051. The out-of-sample prediction of the model was *R*^2^_test_=0.048. (**c**) MPS model predicting body mass index (BMI). Standardized coefficients of polygenic predictors selected by elastic net via repeated cross-validation in training set. Analogous to conventional multiple regression, a standardized coefficient represents the contribution of the predictor to the outcome when adjusting for all other variables in the model. The mean variance explained of the resampling distribution from the cross-validation was mean-cv-*R*^2^_train_=0.074. The out-of-sample prediction of the model was *R*^2^_test_=0.054.

Figures 1a–c show the polygenic predictors selected during training of the MPS models and their standardized coefficients. The ranking of predictors provides an index for their contributions to prediction. Analogous to conventional multiple regression, a standardized coefficient represents the contribution of the predictor to the outcome when adjusting for all other variables in the model.

The model predicting educational achievement retained 12 polygenic predictors (Figure 1a). Cognitive and socioeconomic polygenic scores took the top ranks. However, the psychiatric cross-disorder polygenic score, which aggregates genetic risk for bipolar disorder, schizophrenia, major depressive disorder, autism and attention deficit hyperactivity disorder, and the score for depressive symptoms in the general population were also retained by the model. The scores for Homeostasis Model Assessment of β-cell function, an index of β-cell function, and for coronary artery disease also contributed to prediction of educational achievement.

The MPS model predicting cognitive ability selected 10 polygenic scores during cross-validation (Figure 1b). The strongest contributions to prediction came from cognitive and socioeconomic variables. Contributions from the psychiatric realm came from major depressive disorder, autism spectrum disorder and bipolar disorder, with the latter two having positive association with cognitive ability.

The MPS model predicting BMI retained 28 polygenic scores (Figure 1c). The top three strongest predictions came from obesity-related variables. Ranks four and five were taken by coronary artery disease and age at menarche (negative association). The sixth strongest predictor for children’s BMI was the polygenic score based on the GWAS of mean caudate nucleus volume that plays a role in various non-motor functions including procedural and associative learning and inhibitory action control.^33, 34, 35, 36^ Other predictors included ulcerative colitis, leptin and neuroticism.

### Stratification by MPS

**Figure 2:**
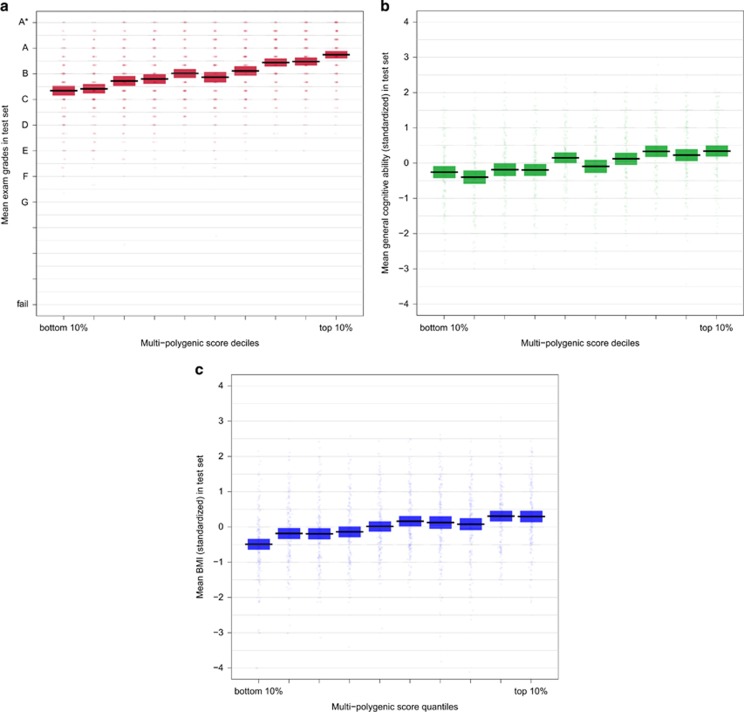
(**a**) Educational achievement by multi-polygenic score (MPS) deciles. Observed mean grade (across the three subjects Mathematics, English and Science) by deciles of the MPS predictions in the test set. Bars represent 95% confidence estimates. (**b**) General cognitive ability by MPS deciles. Observed mean standardized general cognitive ability by deciles of the MPS predictions in the test set. Bars represent 95% confidence estimates. (**c**) Body mass index (BMI) by MPS deciles. Observed mean standardized BMI (age and sex adjusted by external reference) by deciles of the MPS predictions in the test set. Bars represent 95% confidence estimates.

We examined the phenotypic values by quantile of the MPS distribution. Figures 2a–c plot the observed outcomes against the predictions by the MPS model in the test set. In general, the quantile results were roughly linear.

Figure 2a shows quantile results for mean exam grades. Individuals in the top 10% of the MPS distribution on average achieved an ‘A’ mean grade (across the three subjects Mathematics, English and Science), whereas individuals in the bottom 10% MPS distribution achieved a ‘C’ mean grade on average (top 10% mean=9.74; bottom 10% mean=8.33 (11=A*,10=A, 9=B, 8=C, 7=D, 6=E, 5=F, 4=G, 0=failed). Cohen’s *d* was 1.20 (95% confidence interval 0.99–1.41) suggesting that 88% of the top 10% MPS group had a mean grade above that of the bottom 10% group, and there is an 80% probability that a person picked at random from the top 10% MPS group will have a higher score than a person picked at random from the bottom 10% group.^37, 38^

For cognitive ability, Figure 2b illustrates that individuals in the top 10% of the MPS distribution on average had a standardized cognitive ability score over 0.64 (95% confidence interval 0.40–0.89) s.d. higher than those in the bottom 10% MPS distribution. This means that 74% in the top 10% MPS group had mean ability score above that of the bottom 10% group, and that there is a 67% probability that a person picked at random from the top 10% MPS group will have a higher score than a person picked at random from the bottom 10% group.

For BMI, Figure 2c shows that children in the top 10% of the MPS distribution on average had a 0.80 (95% confidence interval 0.57–1.03) s.d. higher than those in the bottom 10% MPS distribution. Expressed differently, 79% of children in the top 10% MPS group had a mean ability score above that of the bottom 10% group, and that there is a 71% probability that a person picked at random from the top 10% MPS group will have a higher score than a person picked at random from the bottom 10% group.

## Discussion

We demonstrate that the MPS approach that combines summary-level GWAS data from multiple traits yields better individual-level phenotype prediction than single-score predictor models in independent test data.

The observation that a multitude of polygenic scores contribute to trait prediction in the MPS models highlights the complexity of the system being studied and the somewhat arbitrary way we divide it into phenotypic characteristics. We show that polygenic variation associated with traits other than the to-be-predicted outcome contributes to prediction. For instance, although there is a known association between ulcerative colitis and BMI,^39^ genetic variants associated with ulcerative colitis are not typically included in models estimating individuals’ genetic risk for increased BMI.

The predictors selected and coefficients estimated by the MPS models in the current study can be used to generate individual-specific composite estimates of genetic propensities in other and smaller samples. For a more parsimonious replication, future research in other samples could construct a simple multiple regression model using the top five predictors selected by the current analyses. The predictive power of such an MPS model can then be compared with that of the best single-score model. More generally, in addition to the likely improvement in MPS prediction as more and larger GWASs are being published, the MPS approach has the potential to be applied to a wide range of outcomes and samples, including psychiatric and medical outcomes in case–control samples.

The predictive power of a polygenic score is not only a function of the genetic correlation between discovery and outcome trait, but also of the statistical power present in the discovery GWAS on which it is based (that is, variance explained and sample size).^5^ The MPS approach exploits the fact that even GWASs of genetically distantly related traits might contribute predictive power if their power is superior to GWASs of more proximal traits. For instance, most likely because of its much greater sample size, the years of education polygenic score predicted general cognitive ability better than any of the polygenic scores based on GWASs directly measuring general cognitive ability.

Because predictive power of polygenic scores does not simply reflect the genetic correlation between discovery and target trait, but depends on the genetic architecture of both traits and sample size (especially of the discovery sample),^5, 6, 40^ the MPS approach is not suited for investigating etiology. Other methods have been developed to that end. For instance, multivariate twin studies are appropriate for investigating trait etiology, or multi-trait GWAS meta-analysis aims to disentangle effects of correlated traits at the level of genetic variants.^15, 16, 41, 42, 43, 44, 45^ In contrast, the declared aim of the MPS approach is to maximize trait prediction, without assumptions about the relationships among predictors.

The MPS approach will be useful whenever trait prediction is a priority. The primary reason for maximizing predictive power using the MPS approach is to predict phenotypes of individuals with as much accuracy as possible. Individual-specific genetic predictions will be useful in research with modest sample sizes to investigate developmental, multivariate and gene–environment interplay issues. Eventually, MPS models could be useful in both society and science to estimate genetic potential as well as risk in relation to all domains of functioning, including cognitive abilities and disabilities, personality and health and illness.

This predictive power will raise concerns about potential early, even prenatal, prediction. It is important to begin discussions that are informed by the empirical data because genotype-based trait prediction is moving towards the point of practical relevance. Although concerns are warranted, these might be outweighed by the benefits that could result from being able to predict problems and potential early and develop stratified preventions and interventions accordingly.
