# Supplementary material for: Multi-polygenic score approach to trait prediction
Source: Mol Psychiatry. 2017 Aug 8;23(5):1368–74. doi: 10.1038/mp.2017.163 (PMC5681246; doi:10.1038/mp.2017.163)
Supplement: Supplementary Information [file mp2017163x1.docx]

**Supplementary information**

**Multi-polygenic score (MPS) approach to trait prediction**

**Supplementary figures**

- Supplementary Figure S1: Phenotypic correlations between target traits
- Supplementary Figure S2: Heatmap singe-score model predictions

**Supplementary tables**

- Supplementary Table S1: Description of GWAS summary statistics
- Supplementary Table S2: Statistics single-polygenic score models

**Supplementary Notes**

- Supplementary Methods S1: Genotyping protocol and quality control
- Supplementary Methods S2: Description of polygenic score creation
- Supplementary Methods S3: Description of outcome measures
- Supplementary References
- Extended Acknowledgements

**Supplementary Figures**

***Supplementary Figure S1: Phenotypic correlations between target traits***

**

*Legend: Supplementary Figure S1*

Phenotypic correlation between composites of children’s Body Mass Index, educational achievement, and general cognitive ability, and their respective subcomponents.

- GCSE core subjects mean: Mean grade of the three core subjects obtained on the standardized United Kingdom General Certificate of Secondary Education (GCSE):
  - GCSE English
  - GCSE Science
  - GCSE Mathematics
- General cognitive ability: Mean of four standardized cognitive test scores:
  - WISC-III-PI Multiple Choice Information (General Knowledge)
  - Vocabulary Multiple Choice subtests
  - WISC-III-UK Picture Completion
  - Raven's Standard and Advanced Progressive Matrices
- Body Mass Index – Age and sex adjusted using external reference

Numbers on the diagonal represent sample sizes for each measure.

***Supplementary Figure S2: Heatmap of singe-score model predictions***

***[see attachment: ‘Supplementary Figure S2.tiff’]***

*Legend Supplementary Figure S2*

Single-polygenic score models (on X-axis) predicting outcomes (body-mas-index; educational achievement; general cognitive ability) in test set. Y-axis represents Gaussian mixture prior used to model the fraction of markers assumed to be causal. The gradient represents standardized coefficients, negative association: green, positive association: pink.

**Supplementary Tables**

***Supplementary Table S1: Results single-polygenic score models***

***[see attachment: ‘Supplementary Table S1.csv’]***

*Legend Supplementary Table S1*

Single-polygenic score models (on X-axis) predicting outcomes (body-mas-index; educational achievement; general cognitive ability).

- Outcome: Outcome trait predicted by model (see Supplementary Methods S3: Description of outcome measures)
- GWAS trait: GWAS summary statistics used to create polygenic score (Supplementary Table S1: Description of GWAS summary statistics; and Supplementary Methods S2: Description of polygenic score creation)
- Gaussian mixture weight: prior used to model the fraction of markers assumed to be causal (Supplementary Methods S2: Description of polygenic score creation)
- mean-cv-Rsquared-train: Mean variance explained from the resampling distribution of the cross-validation in the training set.
- SD-cv-Rsquared-train: Standard deviation of variance explained from the resampling distribution of the cross-validation in the training set.
- mean-cv-RMSE-train: Standardized root-mean-square-error from the resampling distribution of the cross-validation in the training set.
- SD-cv-RMSE-train: Standard deviation of the root-mean-square-error from the resampling distribution of the cross-validation in the training set.
- stdCoeff-test: Standardized coefficient in the test set (see *Supplementary Figure S2: Heatmap of singe-score model predictions for visualization)*
- Rsquared-test: The out-of-sample variance explained (in test set).
- Rsquared-boot95CIlow-test; Rsquared-boot95CIup-test: Bootstrapped (1,000 repeats) confidence intervals of the variance explained in test set.

**Supplementary Notes**

***Supplementary Methods S1: Genotyping protocol and quality control***

DNA for 4,649 individuals was extracted from saliva and buccal cheek swab samples and hybridized to HumanOmniExpressExome-8v1.2 genotyping arrays at the Institute of Psychiatry, Psychology and Neuroscience Genomics & Biomarker Core Facility. The raw image data from the array were normalized, pre-processed, and filtered in GenomeStudio according to Illumina Exome Chip SOP v1.4. (<http://confluence.brc.iop.kcl.ac.uk:8090/display/PUB/Production+Version%3A+Illumina+Exome+Chip+SOP+v1.4>). In addition, prior to genotype calling, 869 multi-mapping SNPs and 353 samples with callrate <.95 were removed. The ZCALL program (see Web resources section) was used to augment the genotype calling for samples and SNPs that passed the initial QC.

DNA from 3,665 samples was extracted from buccal cheek swabs and genotyped at Affymetrix, Santa Clara, California, USA. Samples were successfully hybridized to AffymetrixGeneChip 6.0 SNP genotyping arrays (<http://www.affymetrix.com/support/technical/datasheets/genomewide_snp6_datasheet.pdf>) using experimental protocols recommended by the manufacturer (Affymetrix Inc., Santa Clara, CA). The raw image data from the arrays were normalized and pre-processed at the Wellcome Trust Sanger Institute, Hinxton, UK for genotyping as part of the Wellcome Trust Case Control Consortium 2 (<https://www.wtccc.org.uk/ccc2/>) according to the manufacturer’s guidelines (http://www.affymetrix.com/support/downloads/manuals/genomewidesnp6_manual.pdf). Genotypes for the Affymetrix arrays were called using CHIAMO (https://mathgen.stats.ox.ac.uk/genetics_software/chiamo/chiamo.html).

After initial quality control and genotype calling, the same quality control was performed on the samples genotyped on the Illumina and Affymetrix platforms separately using PLINK(1,2), R (3), and vcftools (4).

Samples were removed from subsequent analyses on the basis of call rate (<0.99), suspected non-European ancestry, heterozygosity, array signal intensity, and relatedness. SNPs were excluded if the minor allele frequency was <0.5%, if more than 1% of genotype data were missing, or if the Hardy Weinberg *p*-value was lower than 10^-5^. Non-autosomal markers and indels were removed. Association between the SNP and the platform, batch, or plate on which samples were genotyped was calculated; SNPs with an effect *p*-value less than 10^-3^ were excluded. A total sample of 6,710 samples, with 3,617 individuals and 600,034 SNPs genotyped on Illumina and 3,093 individuals and 525,859 SNPs genotyped on Affymetrix remained after quality control.

Genotypes from the two platforms were separately imputed using the Haplotype Reference Consortium (5) and Minimac3 1.0.13 (6,7) available on the *Michigan Imputation Server* as reference data. A series of quality checks was performed before merging data from the two platforms’ imputation (e.g. platform effects, allele frequencies by imputation quality). For the present analyses we limited our analyses to variants genotyped or imputed at info >.70 on both platforms, allele frequency difference between platforms smaller than 5%, and Hardy Weinberg *p*-value was greater than 10^-5^. Using these criteria, 7,581,516 genotyped and well-imputed SNPs were retained for the analyses.

We performed principal component analysis on a subset of 42,859 common (MAF>5%) autosomal HapMap3 SNPs (8), after stringent pruning to remove markers in

linkage disequilibrium (*r*^2^> 0.1) and excluding high linkage disequilibrium genomic regions so as to ensure that only genome-wide effects were detected.

***Supplementary Methods S2: Description of polygenic score creation***

We constructed polygenic scores as the weighted sums of the individual’s genotype across all SNPs. We used LDpred (9) to construct the scores. LDpred uses a prior on the markers’ effect sizes and adjusts summary statistics for linkage disequilibrium (LD) between markers.

The polygenic scores were calculated as the weighted sums of individual i’s SNPs:
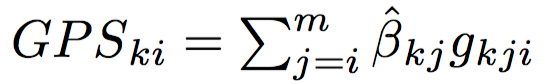


GPS_ik_ represents the individual i’s genome-wide polygenic score based on summary statistics from GWAS_k_. β^_kj_ is an estimate of marker j’s effect size for discovery trait k, that is, the effect of having one more copy of the reference allele at SNP_kj_. g_kji_ is individual i’s genotype at marker j for discovery trait k, coded as having 0,1, or 2 copies of the reference allele at marker _kj_.

Conventionally, the β^_kj_ for SNP_j_ is simply the GWAS k estimate for SNP_jk_. However, due to local linkage disequilibrium (LD) (i.e. correlation) between SNPs, β^_kj_ captures any effects of the SNP_kj_ and its correlates. Therefore, to correct for the multiple counting problem of effectively counting the effects of markers that are in LD with other markers multiple times, conventionally, markers are thinned down via the process of ‘clumping’ to a set of uncorrelated markers prior to polygenic score creation.

In this study, to avoid a reduction in predictive accuracy and loss of information caused by the conventional approach of LD-based marker pruning and applying a P-value threshold to association statistics, we used *LDpred* (9) (version 0.9.09; https://github.com/bvilhjal/ldpred). *LDpred* is a Bayesian approach that infers the posterior mean effect size of each marker by adjusting the effect size from the discovery GWAS using a prior on effect size and information on the LD between the SNPs from a reference panel to obtain a posterior estimate of the causal effect for SNP*_jk_* independent of the effects of other SNPs. Hence, the *LDpred* GPS for individual *i* for GWAS *k* is the sum of *i*’s genotypes across all SNPs used in the analyses, weighted by the *LDpred* estimates of the genotype effects. The score represents an unbiased estimate of the true genetic burden for individual *i* for trait *k* (36), albeit with low precision.

As recommended by the *LDpred* developers (9), we used the target sample genotype data as the LD reference panel. The prior for the effect sizes is a point-normal mixture distribution based on two parameters: the heritability explained by the markers, which is estimated from the GWAS summary statistics, and the fraction of markers assumed to be causal (i.e. fraction of markers with non-zero effects) using a Gaussian mixture weight, which is variable and can be optimized within a validation sample. For each set of GWAS summary statistics, we created *LDpred* scores using Gaussian mixture weights of 1, 0.1, and 0.01.

To account for population stratification, we adjusted the polygenic predictors by the first 30 principal components generated from genotype data prior to the analysis (see Supplementary Methods 1 for details). We used the top 30 PCs as well as genotyping array and plate to create a N*P matrix Z of eigenvectors across the P selected principal components. We then regressed the genetic polygenic predictor onto the eigenvectors as S = μ + Zβ + e, where μ is the mean and β is a P×1vector of the regression coefficients, and e is the residual error. The standardized residuals were entered into the consecutive analyses.

***Supplementary Methods S3: Description of the outcome measures***

*General cognitive ability (g)*

At age 12 individuals were tested on two verbal tests, WISC-III-PI Multiple Choice Information (General Knowledge) and Vocabulary Multiple Choice subtests (10), and two nonverbal reasoning tests, the WISC-III-UK Picture Completion (10) and Raven's Standard and Advanced Progressive Matrices (11,12), all administered online (13,14). *g* scores were derived as the arithmetic mean of the four standardized test scores. We used the residuals after regressing the measure on sex and age at assessment (mean=11.46; SD=0.64) to account for the effects of these variables on *g* (sex: R^2^=0.008, P=8.933e-08; age at assessment: R^2^=0.053; P=<2.2e-16). For this we used the *rstandard*function of the lm package in R (version 3.2.2), which produces standardized residuals via normalization to unit variance using the overall error variance of the residuals.

*Educational achievement*

Educational achievement was operationalized as performance on the standardized United Kingdom General Certificate of Secondary Education (GCSE), taken by almost all (>99%) pupils at the end of compulsory education at age 16. English, mathematics and science are compulsory subjects. Five or more GCSEs with grades A*–C are required for further education, including English and mathematics. The joint performance on the three compulsory subjects determines admission to further education and subsequent employability.

GCSE grades were obtained from the UK National Pupil Database (NPD) (15). Subjects were graded from 4 (G; the minimum pass grade) to 11 (A*; the best possible grade). ‘Ungraded/failed’ exams were coded as 0.

For 1,068 individuals in the sample for whom no NPD data were available, we used self- or parent-reported grades. These data were collected by questionnaires sent by mail and by telephone interview of parents and twins themselves (16). After completed forms were received from the families, the grades were coded from 11 (the highest grade: A*) to 4 (the lowest pass grade: G). No information about the occurrence and number of failed results was available for the self- or parent-reported data. For 9,255 individuals (and 3,584 within the present genotyped sample), self- or parent-reported GCSE results were verified using the data obtained from the UK National Pupil Database (15), yielding correlations of 0.99 for mathematics, 0.98 for English and 0.97 for science.

The GCSE measure for the present analyses was the mean grade of the three compulsory core subjects, mathematics, English (maximum grade of ‘English Language’ and ‘English Literature’), and science (maximum of any science subjects taken). Scores on the three compulsory core subjects were highly correlated (*r* = 0.71–0.79). There was no significant effect of sex on GCSE (R^2^ < 0.001; P=0.051).

*Body Mass Index (BMI)*

When children were aged 8-11 height and weight measurements were obtained as part of a study of the heritability of adiposity (17). Detailed instructions were sent to parents who were asked to record children’s weight and height to the nearest kilogram and centimeter as well as the date of measurement. Researcher-measured and parent-measured weights and heights showed good inter-rater concordance (r =0.90 and r = 0.83) in a subsample of 228 families (17). BMI was calculated as weight (kg)/height (m)^2^ and then converted to z-scores that take into account children’s age and sex using 1990 UK growth reference data (18). This was implemented in the sitar R package (19). Implausible heights (<1.05 or >1.80m), weights (<12 or >80kg), and BMIs (<11 or >32) were excluded based on reference values (18).

**Supplementary References**

1. Chang CC, Chow CC, Tellier LC, Vattikuti S, Purcell SM, Lee JJ. Second-generation PLINK: rising to the challenge of larger and richer datasets. GigaScience. 2015 Feb 25;4(1):7.

2. Purcell S, Neale B, Todd-Brown K, Thomas L, Ferreira MAR, Bender D, et al. PLINK: A Tool Set for Whole-Genome Association and Population-Based Linkage Analyses. Am J Hum Genet. 2007 Sep;81(3):559–75.

3. R Core Team. R: A Language and Environment for Statistical Computing [Internet]. Vienna, Austria: R Foundation for Statistical Computing; 2016. Available from: http://www.R-project.org/

4. Danecek P, Auton A, Abecasis G, Albers CA, Banks E, DePristo MA, et al. The variant call format and VCFtools. Bioinformatics. 2011 Aug 1;27(15):2156–8.

5. McCarthy S, Das S, Kretzschmar W, Durbin R, Abecasis G, Marchini J. A reference panel of 64,976 haplotypes for genotype imputation. bioRxiv. 2015 Dec 23;35170.

6. Howie B, Fuchsberger C, Stephens M, Marchini J, Abecasis GR. Fast and accurate genotype imputation in genome-wide association studies through pre-phasing. Nat Genet. 2012 Aug;44(8):955–9.

7. Fuchsberger C, Abecasis GR, Hinds DA. minimac2: faster genotype imputation. Bioinformatics. 2015 Mar 1;31(5):782–4.

8. Consortium TIH 3. Integrating common and rare genetic variation in diverse human populations. Nature. 2010 Sep 2;467(7311):52–8.

9. Vilhjálmsson BJ, Yang J, Finucane HK, Gusev A, Lindström S, Ripke S, et al. Modeling linkage disequilibrium increases accuracy of polygenic risk scores. Am J Hum Genet. 2015 Jan 10;97(4):576–92.

10. Wechsler D, Golombok S, Rust J. WISC-III UK Wechsler Intelligence Scale for Children: UK manual. Sidcup UK Psychol Corp. 1992;

11. Raven J, Court J, Raven J. Manual for Raven’s progressive matrices and vocabulary scales. Oxford: Oxford University Press; 1996.

12. Raven J, Raven J, Court J. Mill Hill vocabulary scale. Oxford: OPP; 1998.

13. Davis OSP, Haworth CMA, Plomin R. Learning abilities and disabilities: Generalist genes in early adolescence. Cognit Neuropsychiatry. 2009 Jul 1;14(4–5):312–31.

14. Haworth CMA, Harlaar N, Kovas Y, Davis OSP, Oliver BR, Hayiou-Thomas ME, et al. Internet cognitive testing of large samples needed in genetic research. Twin Res Hum Genet. 2007;10(4):554–63.

15. National pupil database - GOV.UK [Internet]. [cited 2014 Sep 10]. Available from: https://www.gov.uk/government/collections/national-pupil-database

16. Shakeshaft NG, Trzaskowski M, McMillan A, Rimfeld K, Krapohl E, Haworth CMA, et al. Strong genetic influence on a UK nationwide test of educational achievement at the end of compulsory education at age 16. PLoS ONE. 2013 Dec 11;8(12):e80341.

17. Wardle J, Carnell S, Haworth CM, Plomin R. Evidence for a strong genetic influence on childhood adiposity despite the force of the obesogenic environment. Am J Clin Nutr. 2008 Feb;87(2):398–404.

18. Cole TJ, Freeman JV, Preece MA. Body mass index reference curves for the UK, 1990. Arch Dis Child. 1995 Jul 1;73(1):25–9.

19. Cole T. sitar: Super Imposition by Translation and Rotation Growth Curve Analysis [Internet]. 2017 [cited 2017 Mar 3]. Available from: https://cran.r-project.org/web/packages/sitar/index.html

**Extended acknowledgements**

We gratefully acknowledge all the studies and databases that made GWAS summary data available: **ADIPOGen** (Adiponectin genetics consortium), **C4D** (Coronary Artery Disease Genetics Consortium), **CARDIoGRAM** (Coronary ARtery DIsease Genome wide Replication and Meta-analysis), **CKDGen** (Chronic Kidney Disease Genetics consortium), **dbGAP** (database of Genotypes and Phenotypes), **DIAGRAM** (DIAbetes Genetics Replication And Meta-analysis), **ENIGMA** (Enhancing Neuro Imaging Genetics through Meta Analysis), **EAGLE** (EArly Genetics & Lifecourse Epidemiology Eczema Consortium, excluding 23andMe), **EGG** (Early Growth Genetics Consortium), **GCAN** (Genetic Consortium for Anorexia Nervosa), **GEFOS** (GEnetic Factors for OSteoporosis Consortium), **GIANT** (Genetic Investigation of ANthropometric Traits), **GIS** (Genetics of Iron Status consortium), **GLGC** (Global Lipids Genetics Consortium), **HaemGen** (haemotological and platelet traits genetics consortium), **HRgene** (Heart Rate consortium), **IIBDGC** (International Inflammatory Bowel Disease Genetics Consortium), **MAGIC** (Meta-Analyses of Glucose and Insulin-related traits Consortium), **PGC** (Psychiatric Genomics Consortium), **ReproGen** (Reproductive Genetics Consortium), **SSGAC (**Social Science Genetics Association Consortium) and **TAG** (Tobacco and Genetics Consortium), **UK Biobank**.

We gratefully acknowledge the contributions of Alkes Price (the systemic lupus erythematosus GWAS and primary biliary cirrhosis GWAS) and Johannes Kettunen (lipids metabolites GWAS).

**Data on glycaemic traits have been contributed by MAGIC investigators and have been downloaded from www.magicinvestigators.org**

Data on coronary artery disease were contributed by CARDIoGRAMplusC4D investigators and were downloaded from <http://www.cardiogramplusc4d.org/>.

We thank the International Genomics of Alzheimer's Project (IGAP) for providing summary results data for these analyses. The investigators within IGAP contributed to the design and implementation of IGAP and/or provided data but did not participate in analysis or writing of this report. IGAP was made possible by the generous participation of the control subjects, the patients, and their families. The i–Select chips was funded by the French National Foundation on Alzheimer's disease and related disorders. EADI was supported by the LABEX (laboratory of excellence program investment for the future) DISTALZ grant, Inserm, Institut Pasteur de Lille, Université de Lille 2 and the Lille University Hospital. GERAD was supported by the Medical Research Council (Grant n° 503480), Alzheimer's Research UK (Grant n° 503176), the Wellcome Trust (Grant n° 082604/2/07/Z) and German Federal Ministry of Education and Research (BMBF): Competence Network Dementia (CND) grant n° 01GI0102, 01GI0711, 01GI0420. CHARGE was partly supported by the NIH/NIA grant R01 AG033193 and the NIA AG081220 and AGES contract N01–AG–12100, the NHLBI grant R01 HL105756, the Icelandic Heart Association, and the Erasmus Medical Center and Erasmus University. ADGC was supported by the NIH/NIA grants: U01 AG032984, U24 AG021886, U01 AG016976, and the Alzheimer's Association grant ADGC–10–196728.

Data on birth length trait has been contributed by EGG Consortium and has been downloaded from www.egg-consortium.org.

A novel common variant in DCST2 is associated with length in early life and height in adulthood. van der Valk RJ, Kreiner-Møller E, Kooijman MN, Guxens M, Stergiakouli E, Sääf A et al. Hum Mol Genet. 2014 Oct 3.

Data on birth weight trait has been contributed by EGG Consortium and has been downloaded from www.egg-consortium.org.

New loci associated with birth weight identify genetic links between intrauterine growth and adult height and metabolism.

Horikoshi M, Yaghootkar H, Mook-Kanamori DO et al. Nat Genet. 2013 Jan;45(1):76-82

Data on head circumference trait has been contributed by EGG Consortium and has been downloaded from [www.egg-consortium.org](http://www.egg-consortium.org).

Common variants at 12q15 and 12q24 are associated with infant head circumference. Taal HR, St Pourcain B, Thiering E, Das S, Mook-Kanamori DO et al. Nat Genet. 2012 Apr;15(4):532-538

Data on the childhood obesity trait has been contributed by EGG Consortium and has been downloaded from www.egg-consortium.org.

A genome-wide association meta-analysis identifies new childhood obesity loci.J.P. Bradfield, H.R. Taal, et al. Nat Genet. 2012 May;44(5):526-31
